# Investigating Ramadan Like Fasting Effects on the Gut Microbiome in BALB/c Mice

**DOI:** 10.3389/fnut.2022.832757

**Published:** 2022-05-12

**Authors:** Junhong Su, Fanglin Li, Yueying Wang, Yuxin Su, Auke Verhaar, Zhongren Ma, Maikel P. Peppelenbosch

**Affiliations:** ^1^Department of Gastroenterology and Hepatology, Erasmus MC – University Medical Center Rotterdam, Rotterdam, Netherlands; ^2^Department of Basic Medicine, Medical School, Kunming University of Science and Technology, Kunming, China; ^3^Engineering and Technology Research Center for Animal Cell, Biomedical Research Center, Northwest Minzu University, Lanzhou, China

**Keywords:** Ramadan, intermittent fasting, BALB/c mice, gut microbiome, human

## Abstract

Recently we reported that in healthy volunteer Ramadan-associated intermittent fasting (RAIF) remodels the gut microbiome and resulted in an increase in small chain fatty acid producing bacteria concomitant with improved metabolic parameters. As interpretation of these results is hampered by the possible psychological effects associated with the study, we now aim to investigate RAIF in experimental animals. To this end, 6-week male BALB/c mice were subjected to RAIF (30 days of a 16-h daily fasting; *n* = 8) or provided with feed *ad libitum* (*n* = 6). Fecal samples were collected before and the end of fasting and bacterial 16S rRNA sequencing was performed. We found that RAIF remodeled the composition of gut microbiota in BALB/c mice (*p* < 0.01) and especially provoked upregulation of butyrate acid-producing Lachnospireceae and Ruminococcaceae (*p* < 0.01), resembling the effects seen in human volunteers. Hence we conclude that the effects of RAIF on gut microbiome relate to the timing of food intake and are not likely related to psychological factors possibly at play during Ramadan.

## Introduction

Intermittent fasting is voluntary temporal partial abstinence or reduction from food consumption, usually showing a daily pattern. As a popular form of temporal dieting, intermittent fasting is widely practiced for a variety of medical, societal, or religious reasons. A growing body of research has shown that intermittent fasting exerts a wide spectrum of impacts on improving indicators of health (including weight management, insulin sensitivity, and inflammation), protecting normal cells or stem cells, delaying aging, and reducing common side effects associated with cancer treatment (1–4).

The most widely practiced form of intermittent fasting is that relating to the Ramadan, in which adherents of the Islamic faith refrain from food and fluid consumption between dawn and sunset for approximately 30 days (5). Recently, we showed that RAIF remodels the gut microbiome in healthy humans, with emphasis on the increase in short-chain fatty acids (SCFAs) producing bacteria. We interpreted these findings as a possible explanation for health effects associated with intermittent fasting (6). As Ramadan is likely to have a plethora of other consequences (psychological and physical) it is possible that the effects observed related to other parameters apart from the timing of food consumption (7). Experimental animals are an obvious way of addressing these concerns and might also be useful for performing mechanistic studies on the effects seen with RAIF.

Prompted by the considerations mentioned above we aimed to establish whether RAIF effects exist in experimental animals. The results show that the effects of RAIF in BALB/c mice resemble those seen in humans, providing support for the notion that spatial timing of food intake *per se* drives the alteration in microbiome composition associated with RAIF in humans.

## Materials and Methods

### Mice and Husbandry Conditions

Male BALB/c Mice (6 weeks old) were randomly grouped into a control group (*n* = 6) and Ramadan model of fasting group (*n* = 8). All mice were individually housed and provided with feed *ad libitum* for 2 weeks prior to study initiation, allowing the animals acclimation to the animal facility. Mice in fasting group were fed every day with 8-h period of free access to food and water followed by 16-h of fasting, and the control group were fed *ad libitum*. The temperature in the animal facility was maintained at approximately 24°C degrees. The water and the diet were sterilized by elevated temperature and irradiation, respectively. Unique fecal samples for each mouse were collected on day 0 and 30. Briefly, mice were individually and temporally placed in a sterilized cage without bedding. Four to five freshly evacuated fecal pellets per mouse were collected in a 1.5 ml sterile tube. All samples were stored at −80°C until further analysis. Animal care was performed according to the Animal Ethics Procedures and Guidelines of the People’s Republic of China, and the experimental protocol was approved by the local committee on animal use and protection, Kunming University of Science and Technology.

### Metagenome Sequencing and Data Processing

Fecal DNA was extracted using the QIAamp DNA Stool Mini Kit (Qiagen, Germantown, MD, United States), according to the manufacturer’s instructions. The DNA quality was monitored on 1% agarose gels. Reagent microbiome and bacterial environmental contamination during DNA library preparation were managed as described elsewhere (6). The quality of the library was assessed using the Qubit@ 2.0 Fluorometer (Thermo Scientific, Waltham, MA, United States). The metagenome sequencing of V3–4 variable regions of the bacterial 16S ribosomal RNA was carried out by the NovoGene Company (Beijing, China). Data processing and analysis were performed according to routine procedures, which have been described elsewhere in detail (8–11).

### Statistical Analysis

Gut microbiome diversity was assessed by the Shannon and Simpson indices, which calculated using alpha-diversity.py in QIIME, and the Mann–Whitney test was used to determine statistical significance between groups. Principal coordinates analysis (PCoA) of Bray–Curtis distance was performed for establishing the shift of the gut microbiota composition after intermittent fasting according to a previously described method (6). Changes in gut microbial shifts were determined by using multivariate data analysis according to analysis of similarities (ANOSIM). A *P*-value of less than 0.05 was considered statistically significant. To identify bacterial taxa whose sequences were differentially abundant between groups, linear discriminant analysis (LDA) coupled with effect size measurements (LEfSe) analysis was applied with the significance level of 0.05 and the logarithmic LDA score threshold equal to 4.^1^

## Results

### Study Execution and Next-Generation Sequencing

**FIGURE 1:**
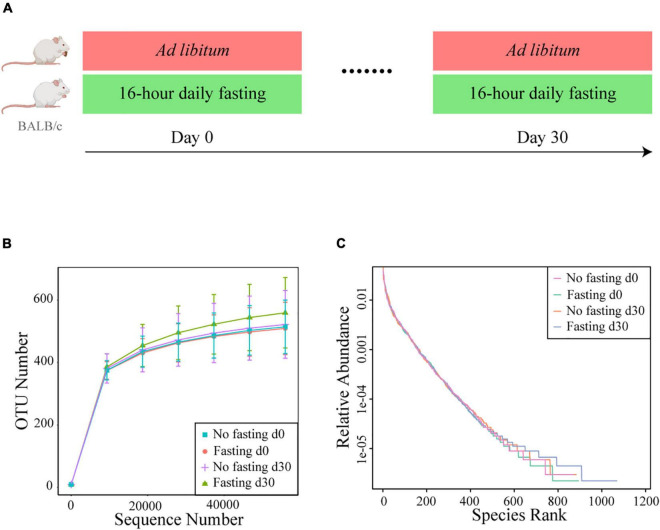
Study design and bacterial 16S rRNA sequencing. **(A)** Experimental design for the 16-h daily fasting group (*n* = 8) and the no fasting group fed *Ad libitum* (*n* = 6). Food and water are only freely available during the other 8 h. The fecal samples were collected at the beginning (Day 0) and end (Day 30) the fasting. After fecal DNA isolation, the bacterial V3-V4 region of 16S rRNA gene was sequenced to map the gut microbiota. The images of mouse model were generated with assistance of Biorender.com. **(B)** Rarefaction curves of detected bacterial OTUs of the gut microbiome from no fasting and fasting groups each reach saturation stage with increasing sequencing depth. Each vertical bar represents standard error. **(C)** The rank abundance curve depicting the bacterial species richness and evenness in the gut microbiome of fasting vs. no fasting mice before or after fasting.

We previously showed in healthy humans that RAIF remodels the gut microbiome and enriches SCFAs-producing microflora, potentially providing a mechanistic explanation for health-promoting effects of intermittent fasting in general and those associated with Ramadan in particular. Further mechanistic studies, however, would require an model in experimental animals, also to avoid the psychological component associated with human studies. In addition such a model would open experimental possibilities not available in human studies, like gnotobiotic experimentation, studies in genetically modified animals and studies independent of the Islamic calendar. Thus prompted, here we aimed to prospectively investigate RAIF effects in BALB/c mice (Figure 1A), generally considered to be a versatile animal model. After fecal DNA isolation, the bacterial V3-V4 region of 16S rRNA gene was sequenced to map the gut microbiota. As a result, a total of 2,287,043 valid sequences were generated and the average for each sample was 68740 ± 4769 (mean ± SD) sequences. Rarefaction curves were plotted to determine the sequence number per group at the same depth. The number of OTUs reached a saturation plateau at a level of 55,000 sequences for each group (Figure 1B), indicating that the sequencing depth was sufficient to represent the majority of bacterial species for each group, although the mice in fasting group show evidence for increased presence of low-abundant bacterial species. Rank-abundance curves were generated to visualize both species richness and evenness of the microbiome in each group at different time points (Figure 1C). There was no clear difference between two groups either before or after IF with respect to species richness and evenness, suggesting that these parameters are not markedly influenced by RAIF in BALB/c mice.

### Intermittent Fasting Alters the Composition of the Gut Microbiome

**FIGURE 2:**
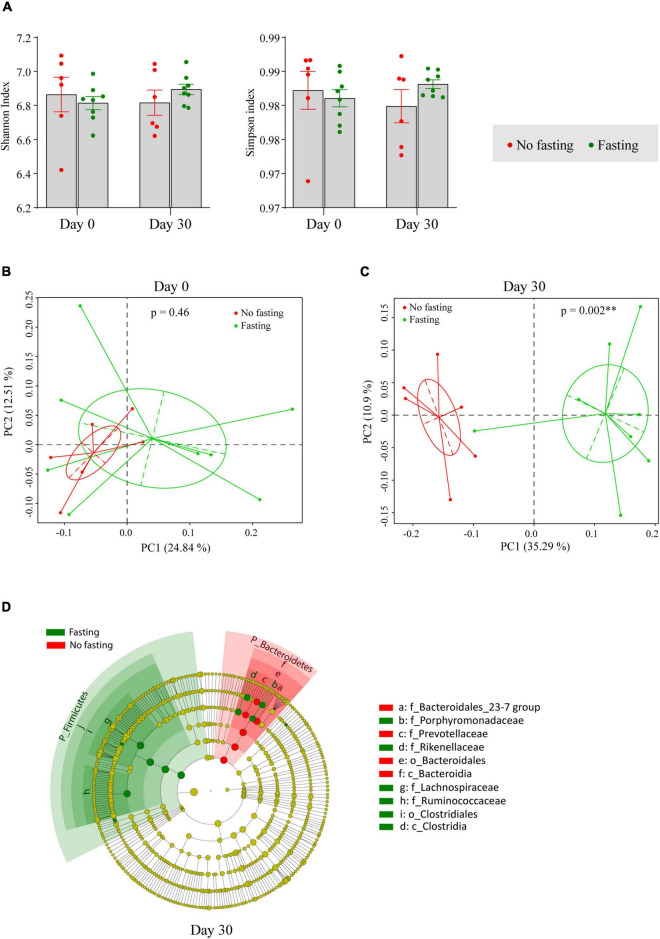
IF shapes the composition of the gut microbiome in BALB/c mice. **(A)** Alpha diversity indexes were shown as Shannon and Simpson. The significance was calculated by unpaired student’s *t*-test. Bray–Curtis distance based Principal Co-ordinates Analysis (PCoA) of the gut microbiome in fasting and no fasting mice at day 0 **(B)** and day 30 **(C)**. Different color represents different groups or time points of sample collection. Each point corresponds to a community from a single individual. Colors indicate community identity. Ellipses show the 95% confidence intervals. ^**^ if *p*-value is less than 0.01 by ANOSIM test. **(D)** Taxa that show alternative abundance before and after fasting are depicted. Taxa with a log linear discriminant analysis (LDA) score above 4.00 as determined by using linear discriminant analysis coupled with effect size measurements (LEfSe). The hierarchy of the discriminating taxonomic levels was visualized as cladograms allowing taxonomic comparisons before and after fasting.

For further analysis of the diversity in the gut microbiome in animals subjected to RAIF and the appropriate controls, the Shannon and Simpson indices, which are important and commonly used diversity indicators of an ecosystem, were calculated. As shown in Figure 2A, gut microbiome diversity was not significantly changed after the fasting, showing that fasting of these animals has no major effect on mean species diversity. Next, changes in microbiome composition were visualized using Bray–Curtis distance-based principal co-ordinates analysis (PCoA). Before the onset of fasting begun (Day 0), microbiome composition was not different between the fasting and non-fasting groups of mice according to Analysis of Similarities (ANOSIM) (*R* = 0, *p* = 0.46) (Figure 2B). After 30 days of IF, however, a remarkable difference was found between the two groups (*R* = 0.74, *p* = 0.002 by ANOSIM) (Figure 2C), indicating that the relative abundance of certain bacterial taxa had changed during the fasting. To further identify the bacterial taxa influenced by fasting, LEfSe analysis was performed. Multiple taxonomic differences were found when comparing the results of fasting mice to the no fasting controls (Figure 2D). At phylum level, an increase of the phylum Firmicutes and depletion of the phylum Bacteroidetes was associated with fasting.

### Intermittent Fasting Elevates the Lachnospiroceae and Ruminococcaceae Families

For further confirmation of the fasting-provoked alterations in the microbiome identified, differences in the bacterial taxa identified by the LEfSe were reanalyzed using unpaired *t*-tests. In agreement with the findings of the LEfSe, we again observed that the phylum Firmicutes was indeed increased as a result of RAIF, while the Bacteroidetes was decreased (Table 1). No significance was found between two groups before fasting. At the family level, it was evident that the abundance of both Lachnospiroceae and Ruminococcaceae was significantly increased by RAIF (Table 1). This may relate to the beneficial effects of IF has on gastrointestinal tract physiology, because these two families are known as important contributors to short-chain fatty acid production in the intestine. Interestingly, the Rikenellaceae and Porphyromonadaceae families were also enriched after the fasting (Table 1). Since upregulation of these two families have been associated with reduced visceral adipose tissue compartment size and improved metabolic profiles in humans (12) and thus the effects seen in mice may reflect a beneficial microbiological shift provoked by IF in general. In contrast, the levels of the Bacteroidetes S24-7 and Prevotellaceae families decreased following fasting as compared to controls, whereas no difference between the groups was found in any of these families before fasting.

**TABLE 1:** Changes in relative abundance of LefSe-identified taxa during fasting.

	*Day*0	Day 30		*P-value*		
			
	Baseline (A) (*n* = 14) Mean ± SD	Non-fasting (B) (*n* = 6) Mean ± SD	Fasting (C) (*n* = 8) Mean ± SD	A vs. B	A vs. C	B vs. C
**Phylum**						
Firmicutes	56.68 ± 9.77	52.79 ± 7.48	67.53 ± 4.84	0.40	0.008	< 0.001
Bacteroidetes	33.32 ± 10.96	38.79 ± 4.93	24.39 ± 6.12	0.26	0.048	< 0.001
**Family**						
Lachnospiraceae	37.46 ± 7.36	34.40 ± 7.36	43.99 ± 3.90	0.41	0.031	0.008
Ruminococcaceae	15.07 ± 2.90	13.98 ± 2.52	19.45 ± 1.99	0.43	0.001	< 0.001
Rikenellaceae	6.64 ± 1.58	5.46 ± 1.19	7.92 ± 1.85	0.12	0.10	0.015
Porphyromonadaceae	2.02 ± 0.48	1.51 ± 0.60	3.88 ± 1.57	0.06	< 0.001	0.005
Bacteroidetes_S24-7_group	20.26 ± 9.64	22.14 ± 3.33	9.77 ± 6.21	0.65	0.012	< 0.001
Prevotellaceae	2.90 ± 2.20	8.63 ± 4.13	2.12 ± 1.44	< 0.001	0.38	0.001

*SD, standard deviation. The significance was calculated by two-sided unpaired student’s t-test.*

## Discussion

Ramadan-associated intermittent fasting is a form of periodic fasting adhered to by millions of adherents of the Islamic faith. A growing number of studies have been conducted in humans to investigate the health effects of RAIF, and we recently characterized the chances in gut microbiome in this respect (6). Volunteer studies in humans suffer, however, from specific cofounders (psychology, altered physical activity during Ramadan, altered circadian rhythms *etcetera*) and studies in humans are less suited for mechanistic investigation (e.g., it is not possible to conduct studies involving genetic modification of the study subjects). A study in experimental animals would show that the effects of RAIF observed indeed relate to the timing of food intake and would allow future mechanistic studies. Prompted by these consideration we characterized the effect of RAIF in BALB/c mice. We observe that changes in microbiome resemble those seen in RAIF in our earlier study (6). The most straightforward interpretation of these observations is that indeed timing of food intake drives the effects seen of RAIF in humans and thus the present study provides further support for the notion that IF can provoke beneficial changes in gut microbiome. In addition, the alterations observed within the bacterial communities might also be provoked by metabolic or immune modifications within host cells such as the production of hormones or ketone bodies, as these factors are known to shape the gut microbiota composition and its function (13, 14). Among the effects, the upregulation of Lachnospiraceae and Ruminococcaceae during the fasting was the most notable and this aligns well with the studies of others associating these bacteria as important drivers for health effects of IF (8, 9).

Over last decade, gut microbiota has attracted great scientific and public attention, and is widely recognized as the nexus of the interaction between life style (e.g., dietary habits) and health status (10). The Lachnospiraceae and Ruminococcaceae families are two most abundant and are seen as the major contributor to luminal biochemistry, while these families are also described as the health-associated core microbiome (11). Both are major SCFAs-producers in our gut and by providing nutritional energy and positional signals to the epithelial compartment play a crucial roles in maintaining the physiological homeostasis of the host. Dysbiosis of these two families is associated with various diseases, especially obesity (15), type 2 diabetes (16), liver cirrhosis (17) and aging (11). Lachnospiraceae are significantly reduced in obesity and levels do not return to those seen in healthy controls even after gastric bypass (15), while Ruminococcaceae were seen to increased following a 2-day modified IF intervention and appear to be negatively associated with the severity of cardiometabolic disease in metabolic syndrome (18). In the present study, we showed that both Lachnospiraceae and Ruminococcaceae families were upregulated in their abundance in the RAIF group, while the abundance of Prevotellaceae was decreased. These changes all echo our previous findings on the alterations of the gut microbiome in healthy subjects who underwent RAIF (6). These data are also in line with previous studies with 7 month of every other day fasting (8, 19). Collectively, this study indicates that experimental rodents can be successfully employed to model RAIF.

Preclinical studies in experimental animals are often essential for understanding disease and therapy and direct design of subsequent clinical study (20). As an extremely important and useful experimental animal, BALB/c mice have been shown to be exceedingly useful for studying both immunology and cancer and many tools tailored for the mouse strain are available (21–23). A great number of studies have been performed to identify the role of the gut microbiome and their implications in health and disease using BALB/C mouse models (22, 23). Successful mimicking the effects of RAIF in this mouse model provides an novel avenue for preclinical studies on how RAIF influences immunology and perhaps cancer progress, especially in gut microbiome dysbiosis-associated conditions.

In summary, we have demonstrated that RAIF shapes the gut microbiota in BALB/c mice, characterized with an increase in SCFAs-producing bacteria. These results support the notion that RAIF in humans works through the temporal spacing of nutrient consumption and provide an experimental model that will allow dissection of the underlying mechanisms.

## Data Availability Statement

The datasets presented in this study can be found in online repositories. The names of the repository/repositories and accession number(s) can be found below: https://www.ncbi.nlm.nih.gov/bioproject/, accession ID: PRJNA789722.

## Ethics Statement

The animal study was reviewed and approved by the Local Committee on Animal Use and Protection, Kunming University of Science and Technology.

## Author Contributions

JS collected and processed the fecal sample, involved in the DNA extraction, performed the PCR reaction, and data analysis. YW, FL, YS, and AV involved in the data interpretation. JS, ZM, and MP involved in the study design and wrote the manuscript. All authors have revised and approved the manuscript.

## Conflict of Interest

The authors declare that the research was conducted in the absence of any commercial or financial relationships that could be construed as a potential conflict of interest.

## Publisher’s Note

All claims expressed in this article are solely those of the authors and do not necessarily represent those of their affiliated organizations, or those of the publisher, the editors and the reviewers. Any product that may be evaluated in this article, or claim that may be made by its manufacturer, is not guaranteed or endorsed by the publisher.

## Footnotes

1
http://huttenhower.sph.harvard.edu/galaxy
